# Cinchonine Prevents High-Fat-Diet-Induced Obesity through Downregulation of Adipogenesis and Adipose Inflammation

**DOI:** 10.1155/2012/541204

**Published:** 2012-05-16

**Authors:** Sung A. Jung, Miseon Choi, Sohee Kim, Rina Yu, Taesun Park

**Affiliations:** ^1^Department of Food and Nutrition, Yonsei University, Seoul 120-749, Republic of Korea; ^2^Department of Food Science and Nutrition, University of Ulsan, Mugeo-dong, Nam-ku, Ulsan 680-749, Republic of Korea

## Abstract

Cinchonine (C_19_H_22_N_2_O) is a natural compound of Cinchona bark. Although cinchonine's antiplatelet effect has been reported in the previous study, antiobesity effect of cinchonine has never been studied. The main objective of this study was to investigate whether cinchonine reduces high-fat-diet- (HFD-) induced adipogenesis and inflammation in the epididymal fat tissues of mice and to explore the underlying mechanisms involved in these reductions. HFD-fed mice treated with 0.05% dietary cinchonine for 10 weeks had reduced body weight gain (−38%), visceral fat-pad weights (−26%), and plasma levels of triglyceride, free fatty acids, total cholesterol, and glucose compared with mice fed with the HFD. Moreover, cinchonine significantly reversed HFD-induced downregulations of WNT10b and galanin-mediated signaling molecules and key adipogenic genes in the epididymal adipose tissues of mice. Cinchonine also attenuated the HFD-induced upregulation of proinflammatory cytokines by inhibiting toll-like-receptor-2- (TLR2-) and TLR4-mediated signaling cascades in the adipose tissue of mice. Our findings suggest that dietary cinchonine with its effects on adipogenesis and inflammation may have a potential benefit in preventing obesity.

## 1. Introduction

Obesity is defined as a phenotypic manifestation of abnormal or excessive fat accumulation that alters health and increases mortality [1]. In obesity phenotypes, the adipose tissue is influenced by diet and genes, as well as by their interactions [2, 3]. Adipocytes are the main cellular component of adipose tissues [4, 5] which is the largest endocrine organ in the body which secretes numerous cytokines and adipokines into the circulation altering body physiology in significant ways [6]. In obese people's adipocytes, both hyperplasia and hypertrophy [7] are observed. The WNT family of autocrine and paracrine growth factors regulates adult tissue maintenance and remodeling and, consequently, is well suited to mediate adipose cell communication [8]. The studies on animals have reported that WNT10b and galanin play important roles in regulating adipogenesis [8]. WNT signaling repressed adipogenesis by blocking induction of peroxisome proliferator-activated receptor *γ* (PPAR*γ*) and CCAAT/enhancer-binding protein *α* (C/EBP*α*) [8]. In high-fat-diet- (HFD-) induced obesity, the expression of WNT10b is decreased, which further reduces the level of *β*-catenin transported to nucleus. *β*-Catenin serves as a cofactor of forkhead transcription factor 1 (FOXO 1) and binds directly to FOXO 1 and enhances FOXO 1 transcriptional activity. FOXO1 competes with T-cell factor (TCF) for interaction with *β*-catenin, thereby inhibiting TCF transcriptional activity and upregulating adipogenesis [9–12]. The HFD also upregulates galanin, its receptors, and some molecules in galanin-mediated signaling pathway such as protein kinase C *δ* (PKC*δ*) and extracellular signal-regulated kinases (ERKs) that induce the expression of target genes of PPAR*γ* and C/EBP*α* [13].

Toll-like receptors (TLRs) are pattern-recognition receptors that detect microbial components and lipopolysaccharides and then activate the immune system, thus providing a first line of host defense against infections [14]. TLR signaling functions through two pathways: a MyD88-dependent pathway and MyD88-independent pathway, both of which trigger the production of proinflammatory cytokines such as interleukin 6 (IL-6) and tumor necrosis factor-*α* (TNF*α*) [15]. In obesity these cytokines likely contribute to the low-level systemic inflammation that is seen in the metabolic syndrome-associated chronic pathologies such as insulin resistance [16–20]. In the HFD model, increased plasma free fatty acids (FFAs) are sensed by TLR2 or TLR4, which activates the nuclear factor kappa-light-chain-enhancer of activated B cells (NF-*κ*B) and in turn induces increased expression of TNF*α*, interleukin 1*β* (IL-1*β*), and IL-6 [21].

Cinchonine (C_19_H_22_N_2_O) is a natural compound found in Cinchona bark, which also contains alkaloids like quinine, quinidine, and cinchonidine. These three agents along with cinchonine have been effectively used as antimalarial drugs [22]. The other unrelated pharmacological effects of these alkaloids include reversal of multidrug resistance in different types of tumors [23]. Cinchonine in this regard has much lower toxicity and higher activity compared to other quinine-related compounds [24]. Previous studies have reported that cinchonine is an inhibitor of human platelet aggregation. Antiplatelet effects of cinchonine are mediated mainly through the inhibition of Ca^2+^ influx and PKC pathways in platelets [25, 26]. Nonetheless, the protective activity of cinchonine against obesity has never been reported. In this study, we investigated the antiobesity effect of cinchonine and its potential mechanism of action on adipocyte differentiation and inflammation using obese C57BL/6 N mice induced by the HFD.

## 2. Materials and Methods

### 2.1. Animal and Diets

Twenty-four male C57BL/6 N mice (Orient, Gyeonggi-do, Korea) were housed in standard cages and placed in a room where the temperature was kept at 21 ± 2.0°C, the relative humidity at 50 ± 5%, and the light at a 12 h light/dark cycle. All the mice consumed a commercial diet and tap water for one week prior to their division into three weight-matched groups (*n* = 8): the normal diet (ND) group, the high-fat diet (HFD) group, and the 0.05% cinchonine-supplemented diet (CID) group. The ND was a purified diet based on the AIN-76 rodent diet composition. The HFD was identical to the ND except that 200 g of fat/kg (170 g of lard plus 30 g of corn oil) and 1% (w/w) cholesterol were added. The CID was identical to the HFD, except that it included 0.05% cinchonine supplementation. The diets were given in the form of pellets for ten weeks.

The mice's food intake was recorded daily, and their body weights were monitored every week during the feeding period. At the end of the experimental period, the animals were anesthetized with ether, following a 12 h period of fasting. Blood was drawn from the inferior vena cava into an ethylene-diamine-tetra-acetic-acid- (EDTA-) coated tube, and the plasma was obtained by centrifuging the blood at 2,000 × g for 15 min at 4°C. Four different locations of visceral fat-pads (epididymal, mesenteric, perirenal, and retroperitoneal) were removed, rinsed with phosphate-buffered saline, and then weighed. The plasma and visceral fat-pad samples were stored at −70°C until analysis. This study adhered to *the Guide for the Care and Use of Laboratory Animals* developed by the Institute of Laboratory Animal Resources of the National Research Council and was approved by the Institutional Animal Care and Use Committee at Yonsei University in Seoul, South Korea.

### 2.2. Histological Analysis

 The epididymal fat-pads were fixed in 10% buffered formalin and embedded in paraffin, cut at thicknesses of 5 *μ*m, and later stained with hematoxylin and eosin (H&E), for the histological examination of adipocyte. Tissue sections were observed with a TDI Digicam camera and mean adipocyte size was determined using TOMORO ScopeEye 3.5 (Techsan Community, Seoul, Korea).

### 2.3. Biochemical Analysis

The plasma concentrations of total cholesterol, HDL cholesterol, triglycerides, glucose, and FFAs were determined enzymatically using commercial kits (Bioclinical System, Gyeonggi-do, Korea). Plasma LDL+VLDL cholesterol levels were calculated by subtracting the HDL cholesterol from the total cholesterol.

### 2.4. Semiquantitative RT-PCR and Quantitative Real-Time RT-PCR Analyses

Total RNA was isolated from the epididymal adipose tissue of each mouse using the Trizol (Invitrogen, Carlsbad, CA, USA) and then analyzed by RT-PCR (*n* = 8). Four micrograms of the total RNA was reverse-transcribed using the Superscript II kit (Invitrogen) according to the manufacturer's instructions. The GenBank accession numbers of the relevant templates and the forward (F) and reverse (R) primer sequences are shown in Table 1. Primers were also designed to amplify a 530-bp cDNA fragment encoding glyceraldehydes 3-phosphate dehydrogenase (GAPDH) as an internal control. The cDNA served as a template in a 40 *μ*L reaction mixture and was processed using an initial step at 94°C for 5 min, followed by 30~33 amplification cycles (94°C for 30 s; 55~60°C for 45 s; 72°C for 1 min) and a final elongation for 10 min at 72°C. Five microliters of each PCR reaction was mixed with 1 *μ*L of six-fold concentrated loading buffer and then loaded onto a 2% agarose gel containing ethidium bromide. Transcript amounts were normalized to GAPDH transcript. Real-time quantitative polymerase chain reaction analyses were performed with cDNA on a LightCycler Instrument (Roche Diagnostics, Basel, Switzerland), using the FastStart DNA Master SYBR Green I (Roche Diagnostics) according to the protocol provided by the manufacturer. Transcript amounts were normalized to GAPDH transcript. PCR products were separated and visualized as described above and band intensities were quantified using Quantity One analysis software (Bio-Rad Laboratories, CA, USA).

### 2.5. Western Blot Analysis

The epididymal adipose tissue samples obtained from each mouse were homogenized in extraction buffer containing 100 mM Tris-HCl, pH 7.4, 5 mM EDTA, 50 mM NaCl, 50 mM sodium pyrophosphate, 50 mM NaF, 100 mM orthovanadate, 1% Triton X-100, 1 mM phenylmethanesulfonyl fluoride, 2 *μ*g/mL aprotinin, 1 *μ*g/mL pepstatin A, and 1 *μ*g/mL leupeptin. Homogenates were centrifuged at 1,300 × g for 20 min at 4°C. The protein concentration of the homogenates was measured by the Bradford assay (Bio-Rad, Hercules, CA, USA). The protein samples were subjected to 8% SDS-PAGE and then electrophoretically transferred to nitrocellulose membranes (Amersham, Buckinghamshire, UK). The nitrocellulose membranes were incubated overnight with primary antibodies (diluted 1 : 1,000) at 4°C. Antibodies to the following proteins were purchased from the indicated sources: ERK, phospho-ERK (Thr 202/Tyr 204), interferon regulatory factor 3(IRF3) and *β*-catenin from Santa Cruz Biotechnology (Santa Cruz, CA, USA), and phospho-IRF3 (Ser 396) and *β*-actin from Cell Signaling Technology (Beverly, MA, USA). After the membranes were incubated with the corresponding secondary antibody, immunoreactive signals were detected using a chemiluminescent detection system (Amersham, Buckinghamshire, UK) and were quantified using Quantity One analysis software (Bio-Rad).

### 2.6. Statistical Analysis

The results are expressed as the mean ± SEM of eight mice in each group. The RT-PCR data were presented as average ± SEM of at least three separate experiments. Statistical significance was calculated using one-way ANOVA, followed by Duncan's multiple range tests. All the statistical analyses were performed with SPSS 12.0 software. Differences were considered statistically significant when *P* < 0.05.

## 3. Results

### 3.1. Body and Visceral Fat-Pad Weights

**Figure 1:**
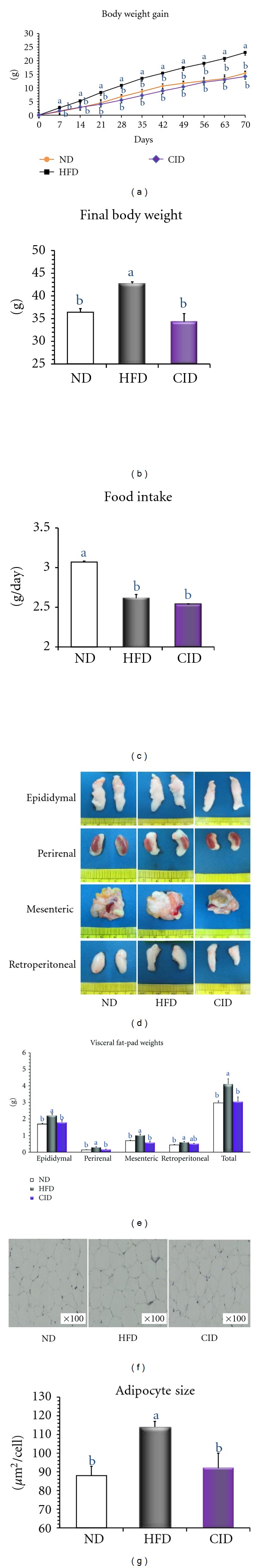
Effect of dietary cinchonine on body weight and fat-pad weights. Mice were fed on ND, HFD, or CID. (a) Changes in body weight gain and (b) final body weight after 10 weeks. (c) Food intake and (d), (e) fat-pad weights. (f) Representative photographs of adipocytes in the epididymal tissue of mice, 100x magnification. (g) Quantitative measurements of adipocyte size (the average cross-sectional area of each adipocyte, um²/cell) in mice fed the ND, HFD, or CID.

Mice fed the HFD had significantly higher body weights than animals fed the ND. Furthermore, dietary supplementation of the HFD with cinchonine at 0.05% (wt/wt) significantly reduced both final body weight (−20%, *P* < 0.05) and body weight gain after 10 weeks of feeding (−38%, *P* < 0.05) compared with the values for HFD mice (Figures 1(a) and 1(b)). There were no statistical differences in food intake among the three different diet groups (Figure 1(c)). The total visceral fat pad weights of mice fed on the HFD, which was significantly greater than the weights of ND mice (by 38%, *P* < 0.05), were reduced when the mice were administered cinchonine (by 26%, *P* < 0.05). The epididymal, perirenal, mesenteric, and retroperitoneal fat-pad weights of the mice given cinchonine were reduced by 19%, 44%, 43%, and 14%, respectively, compared to those of HFD-fed mice (*P* < 0.05) (Figures 1(d) and 1(e)). Histological analysis of adipocyte H&E staining also showed smaller adipocytes in CID-fed mice (−17%) than in HFD-fed mice (Figures 1(f) and 1(g)).

### 3.2. Plasma Biochemistry

**Figure 2:**
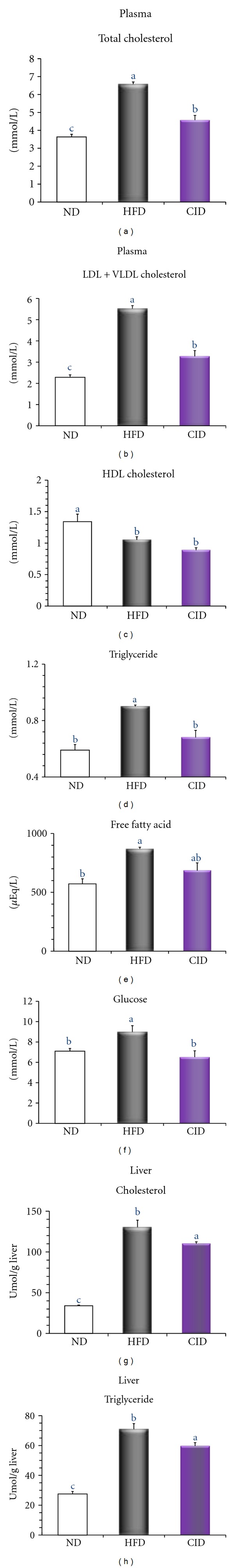
Effects of dietary cinchonine on plasma and liver biomarkers of the mice fed on the ND, HFD, or CID. Plasma (a) total cholesterol, (b) LDL+VLDL-cholesterol, (c) HDL cholesterol, (d) triglycerides, (e) free fatty acids, and (f) glucose levels. Hepatic (g) cholesterol and (h) triglyceride levels. Data are expressed as the mean ± SEM, *n* = 8, *P* < 0.05. Different letters are statistically different.

HFD-induced hypercholesterolemia was significantly improved by dietary supplementation with cinchonine. Plasma concentrations of total and LDL+VLDL cholesterol in mice fed on the CID were significantly decreased by 31% and 41%, respectively, compared to that in the HFD-fed group (Figures 2(a), 2(b) and 2(c)). HFD-induced elevation in the plasma triglyceride and glucose concentrations was reversed when mice were fed on the CID (24% and 27% reduction, respectively, *P* < 0.05). Dietary supplementation with cinchonine tended to decrease plasma FFA levels compared to the levels in HFD control mice; however, this trend did not reach statistical significance (Figure 2(f)). Hepatic levels of cholesterol and triglyceride in mice fed the CID were significantly decreased by 15% and 16%, respectively, compared to that in the HFD-fed mice.

### 3.3. Expression of Adipogenesis-Related Genes

**Figure 3:**
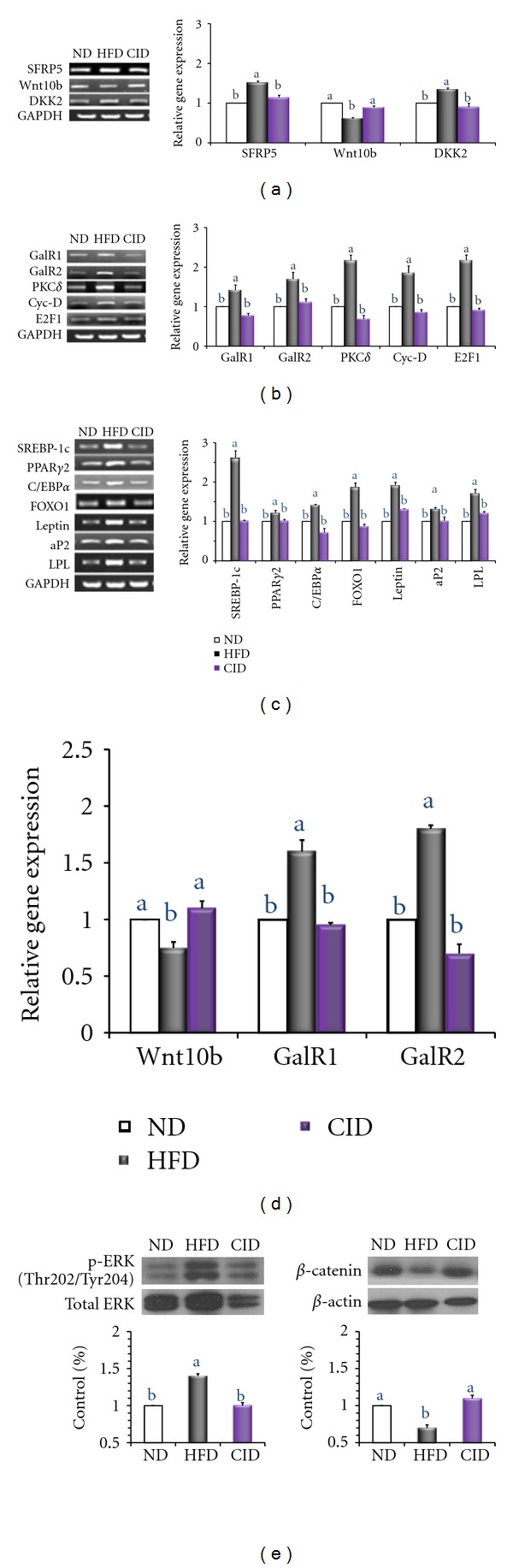
Effect of dietary cinchonine on adipogenic genes. Mice were fed on ND, HFD, or CID. (a) The expression of genes related to adipogenesis was determined by a semiquantitative RT-PCR. (b) Expression of galanin and upstream molecules. (c) Expression of transcription factors and target genes. (d) The expression of Wnt10b, GalR1, and GalR2 was determined by a quantitative real-time RT-PCR. The data shown are the relative density normalized to GAPDH. (e) Protein levels of phosphorylation of ERK (p-ERK), total ERK and total *β*-catenin in the epididymal adipose tissue of mice by Western blot. p-ERK was normalized to their respective total protein level. Total *β*-catenin was normalized to total *β*-actin level. Bars represent the mean ± SEM, *n* = 8,  *P* < 0.05. Different letters are statistically different.

We assessed the impact of the dietary cinchonine on the expression of several genes related to adipogenesis in the epididymal adipose tissue of mice. We found that, compared to HFD-fed mice, CID-fed mice had decreased expression of secreted frizzled-related protein (SFRP) 5 and dickkopf (DKK) 2 and increased expression of WNT10b (Figure 3(a)). Furthermore, CID-fed mice had decreased mRNA levels of galanin receptor 1 (GalR1), galanin receptor 2 (GalR2), PKC*δ*, cyclin D (Cyc-D), and E2F1 (Figure 3(b)). As shown in Figure 3(c), the mRNA levels of PPAR*γ*2, C/EBP*α*, sterol regulatory element-binding protein-1 (SREBP1), and FOXO1 were significantly downregulated in CID mice compared with those in HFD-fed mice (Figure 3(c)). Similar results were observed for the mRNA levels of key adipogenic target genes; expression of leptin, activating protein 2 (aP2) and lipoprotein lipase (LPL) were down-regulated in CID group compared to HFD group. To investigate more subtle differences between groups, we assessed the expression of Wnt10b, GalR1, and GalR2, using a quantitative real-time RT-PCR. In accordance with results obtained by a semiquantitative RT-PCR, we found that, compared to HFD-fed mice, CID-fed mice had increased expression of WNT10b and decreased expression of GalR1 and GalR2 (Figure 3(d)). Moreover, Western blot analysis of proteins confirmed that ERK phosphorylation (Thr202/Tyr204) in CID mice was significantly lower while *β*-catenin level was significantly increased in CID mice compared with that in HFD mice.

### 3.4. Expression of Inflammation-Related Genes

**Figure 4:**
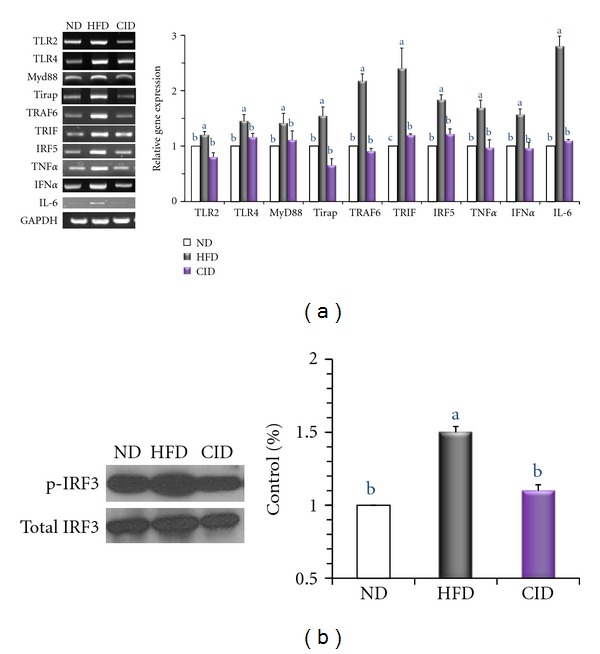
Effect of dietary cinchonine on the expression of genes involved in inflammation. Mice were fed on the ND, HFD, or CID. (a) RT-PCR analyses of TLRs-mediated proinflammatory cytokine genes. The data shown are the relative density normalized to GAPDH. (b) Protein levels of phosphorylation of IRF3 (p-IRF3) and total IRF3 in the epididymal adipose tissue of mice analyzed by Western blot. p-IRF3 was normalized to total IRF3 protein level. Bars represent the mean ± SEM, *n* = 8, *P* < 0.05. Different letters are statistically different.

 We examined whether cinchonine can attenuate HFD-induced activation of TLR-mediated proinflammatory signaling in the epididymal adipose tissue of mice. RT-PCR analysis confirmed the elevated expression of TLR2, TLR4, myeloid differentiation primary response gene 88 (MyD88), toll-interleukin 1 receptor domain-containing adaptor protein (Tirap), TNF receptor-associated factor 6 (TRAF6), and TIR-domain-containing adapter-inducing interferon-*β* (TRIF) in HFD-fed mice (Figure 4(a)). As shown in Figure 4(a), the mRNA levels of pro-inflammatory transcription factors (IRF5) and target cytokines genes (TNF*α*, interferon *α* (IFN*α*), and IL-6) were upregulated in HFD group compared to those in ND group. Our data indicate that cinchonine supplementation successfully abolished HFD-induced upregulation in the expression of inflammation-related genes. In CID group, the expression of TLR2, TLR4, Tirap, TRAF6, IRF5, TNF*α*, and IFN*α* was approximately 1.5-fold lower and MyD88 and TRIF expression was 2-fold lower than that of HFD group. The protein levels of IRF3 and phospho-IRF3 in the epididymal tissue of mice were determined by Western blot analysis, and the results showed 29% higher IRF3 phosphorylation in HFD mice when compared to that in CID mice.

## 4. Discussion

 Despite the growing obese population worldwide, pharmacotherapy for obesity is limited. In 2010, FDA withdrew sibutramine from the market due to its association with increased cardiovascular events and strokes, making orlistat the one and only drug for obesity. However, new concerns of potential liver toxicity with orlistat have recently been raised, further reducing the armamentarium for the combat of obesity [27]. Therefore, taking into account the limited efficacy and uncertain safety of the available drugs, an emphasis should be placed on developing pharmacologic agents with novel mechanisms for decreasing adipogenesis and inflammation to enhance efficacy and improve safety.

 In acute toxicity study, maximal tolerated i.p. dose of cinchonine was 200 mg/kg body in mice [28]. Cinchonine is structurally similar to quinine which is one of the major alkaloids isolated from the Cinchona bark. Previous study showed that, in pentylenetetrazole seizure model, quinine (60 mg/kg BW, i.p.) significantly inhibited both induction and duration of seizure [29]. Based on the results of previous studies, 0.05% (equivalent to 50 mg/kg body weight) cinchonine supplementation was used in the present study.

Until now, there were no studies on cinchonine's antiobesity effect. Our study showed that cinchonine could be a potential agent that can solve the concerns related to obesity. Cinchonine demonstrated more dramatic effect than other phytochemicals that have been known to exert anti-obesity effects; 0.05% cinchonine showed higher rate of reductions compared to EGCG and curcumin in final body weight even though the supplemented dose was higher than or same as that of cinchonine [30, 31]. It was reported that 0.32% EGCG supplemented group showed 9.4% decrease in final body weight compared to HFD fed mice [32]. 0.05% curcumin supplementation is also known to lower the body weight by 11% in the same model [31]. Along with cinchonine's effect on body weight reduction, cinchonine decreases the plasma level of lipid in mice fed on the HFD. Cinchonine effectively ameliorated hyperlipidemia and hyperglycemia induced by the HFD; cholesterol, LDL+VLDL cholesterol, HDL cholesterol, TG, and the plasma glucose levels were reduced in CID group compared to HFD group. Cinchonine treatment blunted the HFD-mediated hyperlipidemia and hyperglycemia that are early symptoms of the metabolic syndrome and associated disorders.

 PPAR*γ*, a member of the nuclear receptor subfamily of transcription factors, is involved in the expression of target genes implicated in adipocyte differentiation [32]. Even though WNT10b and galanin signal through different pathways, they regulate adipogenesis by eventually altering the expression of PPAR*γ*2 and adipogenic regulators. WNT, a family of secreted glycoproteins, blocks the induction of PPAR*γ*2 and C/EBP*α* which further repress the adipogenesis through inhibiting the subsequent nuclear translocation of *β*-catenin [8]. Galanin, a neuropeptide with 29-30 amino acids, binds to GalR1 and GalR2 resulting in the activation of PKC*δ*. Subsequent activation of PKC*δ* can induce the activation of protein tyrosine kinases that could lead to ERK activation [33] which is necessary for expression of the crucial adipogenic regulators and PPAR*γ*.

**Figure 5:**
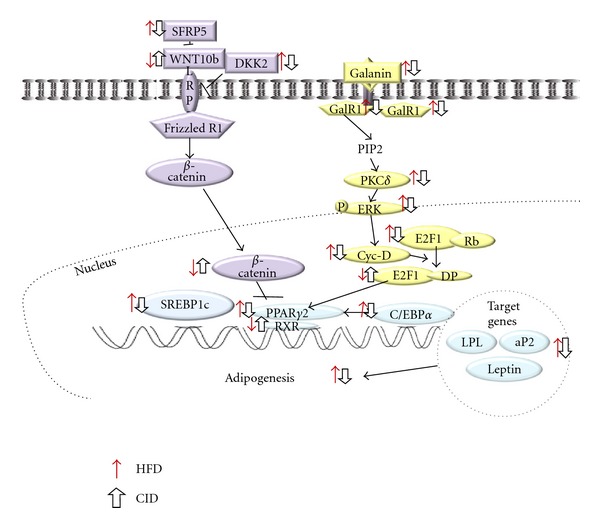
Schematic of the WNT10b and galanin-mediated signaling pathways linked to adipogenesis. In HFD model, increased SFRP5 and DKK2 inhibit WNT10b to combine with LRP and frizzed receptor. Decreased WNT10b signal transduction increases the phosphorylation of *β*-catenin thus reducing the amount of *β*-catenin transported to nucleus. In HFD model, decreased amount of *β*-catenin in nucleus cannot inhibit the activity of PPAR*γ* resulting in the stimulation of adipogenesis. The HFD also increases gene expression in the galanin-mediated signaling pathway. More galanin binds to its receptors, resulting in increased phosphorylation of ERK, which further induces Cyc-D. Increased Cyc-D promotes more E2F1 to bind to DP that stimulates the expression of PPAR*γ*2, C/EBP*α*, and its target genes.

 The HFD has the capacity to modulate both WNT10b and galanin signaling pathways in adipogenesis (Figure 5) [34]. Our results demonstrated the decreased expression of WNT10b and *β*-catenin, and the increased expression of galanin, its receptors and down-stream molecules including PPAR*γ*2 in HFD-fed mice. In the present study, cinchonine supplementation significantly reversed the HFD-induced elevations of adipogenic genes involved in both the WNT and galanin-mediated signaling pathways. By markedly upregulating the expression of WNT10b and downregulating GalRs, cinchonine consequently reduced the expression of PPAR*γ*2 and its target genes (C/EBP*α*, leptin, aP2, and LPL). Besides regulating the WNT and galanin-mediated signaling pathway, cinchonine might further repress the activation of PPAR*γ* by reducing the plasma FFA level which is known as an exogenous ligand for PPAR*γ*. Cinchonine's effect on the reduction of visceral fat-pad weights is presumed to be through altering the WNT and galanin-mediated signaling pathways, and also through lowering plasma FFA level. Furthermore, in several reports by other investigators, nutrient excess activates biochemical pathways that initiate cellular responses designed to limit the oxidation of excess energy and favor weight gain. Simultaneously, the activation of the same pathways either directly or indirectly induces the dissipation of excess energy via thermogenesis [35]. Accordingly, further studies are required to evaluate the possible roles of cinchonine in fatty acid oxidation and thermogenesis pathways.

FFA is also an endogenous ligand for TLR2 and TLR4 which play important roles in the pathogenesis of noninfectious, inflammatory diseases of host deregulation such as obesity [36–38]. Mice fed on the HFD have elevated level of FFAs, further inducing the expressions of TLR2 and TLR4. Of two different inflammatory signaling pathways, TLR4 uses both MyD88-dependent and MyD88-independent pathways, whereas TLR2 signals only through a MyD88-dependent pathway [22]. The increased interaction and recruitment of adaptor molecules such as MyD88, Tirap, TRIF, and TRAM with TLRs in HFD model trigger the induction of TRAF6 in the MyD88-dependent pathway [15]. Then, TRAF6 activates IRF5 leading to its nuclear translocation and cooperation with NF-*κ*B [15]. In the MyD88-independent pathway, elevated TRIF promotes the phosphorylation of IRF3 [15]. Both IRF5 and IRF3 stimulate the expression of target genes such as IL-6, TNF*α*, and IFN*α*, which act on the TNF receptor in hypertrophied adipocytes, thereby inducing pro-inflammatory cytokine production via NF-*κ*B-dependent and -independent mechanisms [39]. Dietary cinchonine successfully reduced the mRNA levels of TLR2, TLR4, downstream molecules (MyD88, Tirap, TRIF, TRAF6, IRF5, and IRF3), and pro-inflammatory cytokines (TNF*α* and IFN*α*) in the epididymal adipose tissue of mice fed on the HFD. In the present study, the plasma concentration of FFA decreased by cinchonine could also be a factor in the reduction of obesity-induced inflammation. Accordingly, we presume that cinchonine contributes to lower adipose inflammation in the diet-induced obese model by down-regulating the TLR-mediated signaling pathway and lowering plasma FFA level in mice maintained on the HFD.

In conclusion, our results indicate that cinchonine has a dramatic suppressive effect on adipogenesis through the down-regulation of WNT and galanin-mediated adipogenesis signaling cascades, and it also attenuates inflammation by repressing TLR2- and TLR4-mediated pro-inflammatory signaling pathways in the adipose tissue of mice fed on the HFD. In the current study, we demonstrated that cinchonine is a useful dietary phytochemical for the prevention of not only obesity, but also adipose inflammation.

## Figures and Tables

**Table 1:** Primer sequences and PCR conditions.

Gene description	Primers	Sequences (5′→3)	Annealing temperature(°C)	PCR product (bp)
Peroxisome proliferator-activated receptor gamma2 (PPAR*γ*2)	F R	TTCGGAATCAGCTCTGTGGA CCATTGGGTCAGCTCTTGTG	55	148
CCAAT/enhancer-binding protein, alpha (C/EBP*α*)	F R	AAGGCCAAGAAGTCGGTGGA CCATAGTGGAAGCCTGATGC	55	189
Adipocyte protein 2 (aP2)	F R	ACATGAAAGTGGGAGTG AAGTACTCTCTGACCGGATG	55	128
Leptin	F R	CTCCAAGGTTGTCCAGGGTT AAAACTCCCCACAGAATGGG	55	143
Sterol regulatory element-binding factor 1 (SREBP1c)	F R	TTGTGGAGCTCAAAGACCTG TGCAAGAAGCGGATGTAGTC	55	94
Lipoprotein lipase (LPL)	F R	TGCCGCTTTTTGTTTTACC TCACAGTTTCTGCTCCCAGC	55	172
Secreted frizzled-related protein 5 (SFRP5)	F R	CTTGGTGTCCTTGCGCTTTA CTGATGGCCTCATGGAACAG	61	155
Wingless-type MMTV integration site family, member 10B (WNT10b)	F R	TTTTGGCCACTCCTCTTCCT TCCTTTTCCAACCGAAAACC	61	183
Dickkopf 2 (DKK2)	F R	GCATTTCCTTCAGATTGGCA TCATTCCCTGTTCTTCAGCG	55	144
Galanin receptor 1 (GalR1)	F R	CCAAGGGGGTATCCCAGTAA GGCCAAACA CTACCAGCGTA	55	147
Galanin receptor 2 (GalR2)	F R	ATAGTGGTGCTCATGCTGGAA AGGCTGGATCGAGGGTTCTA	60	134
Protein kinase C *δ* (PKC*δ*)	F R	CTGAGCGCTGCAAGAAGAAC TGGAAACTTTGATCCTGCACTGA	60	146
Cyclin D (Cyc-D)	F R	TGGGAAGTTTTGTTGGGTCA TCCTTGTCCAGGTAATGCCA	55	144
E2F1	F R	CCTGGAGCATGTTAAAGAAG CCTCGAGACCAAAGTGATAG	48.9	102
Toll-like receptor 2 (TLR2)	F R	TCTAAAGTCGATCCGCGACA TACCCAGCTCGCTCACTACG	56.2	344
Toll-like receptor 4 (TLR4)	F R	ACCTCTGCCTTCACTACAGA AGGGACTTCTCAACCTTCTC	48.6	223
Myeloid differentiation primary response gene 88 (MyD88)	F R	AAGAAAGTGAGTCTCCCCTC TCCCATGAAACCTCTAACAC	55	149
Toll-interleukin 1 receptor domain-containing adaptor protein (Tirap)	F R	GTGGCCGCTGGAGCAAAGAC TTGCCTCTGCCATCCACATA	55	370
TNF receptor-associated factor 6 (TRAF6)	F R	GCACAAGTGCCCAGTTGACA AGTGTCGTGCCAAGTGATTC	62.7	479
TIR-domain-containing adapter-inducing interferon-*β* (TRIF)	F R	ATGGATAACCCAGGGCCTT TTCTGGTCACTGCAGGGGAT	56.5	528
Interferon regulatory factor 5 (IFR5)	F R	AATACCCCACCACCTTTTGA TTGAGATCCGGGTTTGAGAT	53.1	191
Interferon *α* (IFN*α*)	F R	ATGGCTAG(G/A)CTCTGTGCTTTCCT GGGCTCTCCAGA(T/C)TTCTGCTCTG	60.2	500
Tumor necrosis factor alpha (TNF*α*)	F R	TGTCTCAGCCTCTTCTCATT AGATGATCTGAGTGTGAGGG	55	156
Intereukin 6 (IL-6)	F R	TTGCCTTCTTGGGACTGATG CCACGATTTCCCAGAGAACA	55	162
Forkhead box transcription factor O1 (FOXO1)	F R	TCCCAATGGCACAGTCCTTA AGCAGTCCAAAGATGCCCTT	55.2	185
Glyceraldehyde-3-phosphate dehydrogenase (GAPDH)	F R	AGAACATCATCCCTGCATCC TCCACCACCCTGTTGCTGTA	60	321

## References

[1] Gonzalez-Castejon M, Rodriguez-Casado A (2011). Dietary phytochemicals and their potential effects on obesity: a review. *Pharmacological Research*.

[2] Ordovas JM, Shen J (2008). Gene-environment interactions and susceptibility to metabolic syndrome and other chronic diseases. *Journal of Periodontology*.

[3] Marti A, Martinez-Gonzalez MA, Martinez JA (2008). Interaction between genes and lifestyle factors on obesity. *Proceedings of the Nutrition Society*.

[4] Weisberg SP, McCann D, Desai M, Rosenbaum M, Leibel RL, Ferrante AW (2003). Obesity is associated with macrophage accumulation in adipose tissue. *Journal of Clinical Investigation*.

[5] Xu H, Barnes GT, Yang Q (2011). Chronic inflammation in fat plays a crucial role in the development of obesity-related insulin resistance. *Journal of Clinical Investigation*.

[6] Ouchi N, Parker JL, Lugus JJ, Walsh K (2011). Adipokines in inflammation and metabolic disease. *Nature Reviews Immunology*.

[7] Jo J, Gavrilova O, Pack S (2009). Hypertrophy and/or hyperplasia: dynamics of adipose tissue growth. *PLoS Computational Biology*.

[8] Christodoulides C, Lagathu C, Sethi JK, Vidal-Puig A (2009). Adipogenesis and WNT signalling. *Trends in Endocrinology and Metabolism*.

[9] Manolagas SC, Almeida M (2007). Gone with the Wnts: *β*-catenin, T-cell factor, forkhead box O, and oxidative stress in age-dependent diseases of bone, lipid, and glucose metabolism. *Molecular Endocrinology*.

[10] Essers MAG, de Vries-Smits LMM, Barker N, Polderman PE, Burgering BMT, Korswagen HC (2005). Functional interaction between *β*-catenin and FOXO in oxidative stress signaling. *Science*.

[11] Hoogeboom D, Essers MAG, Polderman PE, Voets E, Smits LMM, Burgering BMT (2008). Interaction of FOXO with *β*-catenin inhibits *β*-catenin/T cell factor activity. *The Journal of Biological Chemistry*.

[12] Munekata K, Sakamoto K (2009). Forkhead transcription factor Foxo1 is essential for adipocyte differentiation. *In Vitro Cellular and Developmental Biology—Animal*.

[13] Kim A, Park T (2010). Diet-induced obesity regulates the galanin-mediated signaling cascade in the adipose tissue of mice. *Molecular Nutrition and Food Research*.

[14] Kawai T, Akira S (2010). The role of pattern-recognition receptors in innate immunity: update on toll-like receptors. *Nature Immunology*.

[15] Fresno M, Alvarez R, Cuesta N (2011). Toll-like receptors, inflammation, metabolism and obesity. *Archives of Physiology and Biochemistry*.

[16] Schenk S, Saberi M, Olefsky JM (2008). Insulin sensitivity: modulation by nutrients and inflammation. *Journal of Clinical Investigation*.

[17] Hotamisligil GS (2006). Inflammation and metabolic disorders. *Nature*.

[18] Berg AH, Scherer PE (2005). Adipose tissue, inflammation, and cardiovascular disease. *Circulation Research*.

[19] Rocha VZ, Libby P (2009). Obesity, inflammation, and atherosclerosis. *Nature reviews. Cardiology*.

[20] Matsuzawa Y, Funahashi T, Nakamura T (1999). Molecular mechanism of Metabolic Syndrome X: contribution of adipocytokines-adipocyte-derived bioactive substances. *Annals of the New York Academy of Sciences*.

[21] Cho S, Choi Y, Park S, Park T (2011). Carvacrol prevents diet-induced obesity by modulating gene expressions involved in adipogenesis and inflammation in mice fed with high-fat diet. *Journal of Nutritional Biochemistry*.

[22] Tracy JW, Webster LT (1996). Drugs used in the chemotherapy of protozoal infections. *The Pharmacological Basis of Therapeutics*.

[23] Genne P, Duchamp O, Solary E (1994). Comparative effects of quinine and cinchonine in reversing multidrug resistance on human leukemic cell line K562/ADM. *Leukemia*.

[24] Genne P, Duchamp O, Solary E, Magnette J, Belon JP, Chauffert B (1995). Cinchonine per os: efficient circumvention of P-glycoprotein-mediated multidrug resistance. *Anti-Cancer Drug Design*.

[25] Shah BH, Safdar B, Virani SS, Nawaz Z, Saeed SA, Gilani AH (1997). The antiplatelet aggregatory activity of *Acacia nilotica* is due to blockade of calcium influx through membrane calcium channels. *General Pharmacology*.

[26] Gilani AH, Shaheen F Studies on dual antihypertensive activity of cinchonine: an alkaloid from cinchona bark.

[27] Kablan A, Saunders RA, Szkudlarek-Mikho M (2010). Prieurianin causes weight loss in diet-induced obese mice and inhibits adipogenesis in cultured preadipocytes. *Journal of Diabetes & Metabolism*.

[28] Genne P, Dimanche-Boitrel MT, Mauvernay RY (1992). Cinchonine, a potent efflux inhibitor to circumvent anthracycline resistance in vivo. *Cancer Research*.

[29] Nassiri-Asl M, Zamansoltani F, Torabinejad B (2009). Antiepileptic effects of quinine in the pentylenetetrazole model of seizure. *Seizure*.

[30] Sae-Tan S, Grove KA, Kennett MJ, Lambert JD (2011). (−)-Epigallocatechin-3-gallate increases the expression of genes related to fat oxidation in the skeletal muscle of high fat-fed mice. *Food and Function*.

[31] Ejaz A, Wu D, Kwan P, Meydani M (2009). Curcumin inhibits adipogenesis in 3T3-L1 adipocytes and angiogenesis and obesity in C57/BL mice. *Journal of Nutrition*.

[32] Desvergne B, Wahli W (1999). Peroxisome proliferator-activated receptors: nuclear control of metabolism. *Endocrine Reviews*.

[33] Hawes JJ, Narasimhaiah R, Picciotto MR (2006). Galanin and galanin-like peptide modulate neurite outgrowth via protein kinase C-mediated activation of extracellular signal-related kinase. *European Journal of Neuroscience*.

[34] Little TJ, Horowitz M, Feinle-Bisset C (2007). Modulation by high-fat diets of gastrointestinal function and hormones associated with the regulation of energy intake: implications for the pathophysiology of obesity. *American Journal of Clinical Nutrition*.

[35] Obici S, Rossetti L (2003). Minireview: nutrient sensing and the regulation of insulin action and energy balance. *Endocrinology*.

[36] Fessler MB, Rudel LL, Brown JM (2009). Toll-like receptor signaling links dietary fatty acids to the metabolic syndrome. *Current Opinion in Lipidology*.

[37] Shi H, Kokoeva MV, Inouye K, Tzameli I, Yin H, Flier JS (2006). TLR4 links innate immunity and fatty acid-induced insulin resistance. *Journal of Clinical Investigation*.

[38] Song MJ, Kim KH, Yoon JM, Kim JB (2006). Activation of Toll-like receptor 4 is associated with insulin resistance in adipocytes. *Biochemical and Biophysical Research Communications*.

[39] Suganami T, Tanimoto-Koyama K, Nishida J (2007). Role of the Toll-like receptor 4/NF-*κ*B pathway in saturated fatty acid-induced inflammatory changes in the interaction between adipocytes and macrophages. *Arteriosclerosis, Thrombosis, and Vascular Biology*.

